# Quality of antenatal care services at public health facilities of Bahir-Dar special zone, Northwest Ethiopia

**DOI:** 10.1186/1472-6963-13-443

**Published:** 2013-10-26

**Authors:** Tadese Ejigu, Mirkuzie Woldie, Yibeltal Kifle

**Affiliations:** 1 Department of Public Health, Bahir- Dar University, Bahir Dar, Ethiopia; 2 Department of Health Planning and Management, Jimma University, Jimma, Ethiopia

**Keywords:** Antenatal care, Quality of health care, Public health facilities, Prenatal care

## Abstract

**Background:**

Antenatal care (ANC) is one of the evidence based interventions to decrease the probability of bad health outcomes for mothers and their newborns. Effectiveness of antenatal care, however, relies on the quality of care provided during each antenatal care visit. Hence this study attempted to assess the quality of antenatal care services at public health facilities of Bahir-Dar special zone, North Western Ethiopia.

**Methods:**

A facility based cross-sectional study employing both quantitative and qualitative methods was conducted from March to April 2010 in Bahir-Dar special zone, North Western Ethiopia. Quality of care was measured as a proportion of patients receiving recommended components of care. To measure the indicators, data was collected from 369 pregnant women who attended ANC clinics in eight public health facilities, during the data collection period. Data were collected through exit interviews with ANC attendees, observation during consultation, and in-depth interviews with health care providers.

**Results:**

Pregnant mothers attending ANC clinics were found to receive only part of recommended care components. Venereal Disease Research Laboratory (VDRL) test, blood group and Rhesus factor tests were done only for 73 (19.8%) and 133 (36.0%) of the women, respectively. Moreover 236 (64.0%) of the mothers missed the opportunity of receiving iron/folic acid supplement during their ANC visit. Three hundred fifty five (96.2%) of the women received tetanus toxoid vaccine. And only 226 (61.2%) of the women had their conjunctiva checked for anemia. Lack of reagents partly explained the problems observed in the provision of recommended care components.

**Conclusion:**

Almost half, 175 (47.7%) of the study women were not satisfied and a large proportion of mothers are missing opportunities to receive screening (like blood pressure and weight measurements) and preventive components of antenatal care (iron/folic acid supplementation). Therefore, efforts should be targeted to avoid missed opportunities by taking quality improvement measures including the fulfillment of all necessary resources.

## Background

Every pregnancy carries a risk of complications and some pregnancies carry more risks than others. However, many complications occur among women deemed to have less risk and even those women identified to be at higher risk give birth with no complications. As a result, recent researches in the area tend to recommend considering all pregnancies as risky [1].

For normal pregnancies, WHO recommends only four antenatal visits with the first visit in the first trimester (ideally before 12 weeks but no longer than 16 weeks), at 24–28 weeks, 32 weeks and 36 weeks. Each visit should include care that is appropriate to the woman’s overall condition and stage of pregnancy and help her preparing for birth and care for the newborn. If problems or potential problems that will affect the pregnancy and newborn are detected the frequency and scopes of visits are increased. Therefore, the major goal of focused antenatal care (FANC) is to help women maintain normal pregnancies through identification of pre-existing health conditions, early detection of complications arising during pregnancy, health promotion and disease prevention and birth preparedness and complication readiness planning [2,3].

All pregnant women should receive immunization against tetanus and iron and folate supplementation. In addition, every woman should have a plan for a skilled attendant at birth, the place of birth and how to get there, items needed for the birth, money saved to pay the skilled provider and for any needed medications and supplies [3]. However, studies have shown that there are many missed opportunities for care, both because of client- and health system-related factors. Mothers and children may face risks because of limited or close to term ANC visits, low-quality care during visits due to poor provider training, infrastructure and administrative weakness at facilities, complications of existing conditions such as TB, malaria, anemia, or sexually transmit-ted infections (STIs), and short intervals between births [4]. The new approach to ANC also emphasizes the quality of care rather than the quantity [5].

Despite the fact that quality antenatal care is essential for further improvement of maternal and child health, the quality of ANC service is not well studied in Ethiopia in general and the Amhara Region in particular. Therefore the objective of this research is to assess the quality of antenatal services and its associated factors in public health facilities of Bahir-Dar city Administration by the year 2010 using primary data that will be collected from the study area and the output of this study could be used for improvement of ANC services delivered by the health system of the study area in particular and other similar setting.

## Methods

### Study setting

A facility based cross-sectional study was conducted in Bahir-Dar Special Zone from March and April 2010. The study was conducted in eight government owned ANC clinics (Felge- Hiwot referral hospital, Bahir Dar HC, HanHC, Abay-mado HC, Meshenti Hc, Zenzelma HC, Tis Abay HC, and Zegie HC). Antenatal care is one of the preventive and Promotive health care services being provided in each of the health facilities. The service is provided by either male or female midwives or clinical nurses in all health facilities. The clinics primarily serve pregnant women from Bahir Dar Special Zone. The Zone is one of the eleven zones in the Amhara Regional State, which is located in the North Western part of Ethiopia. According to the Ethiopian National census report in 2007, the total population of the Zone is 220, 334; of this 180,094 (81.7%) people are urban dwellers while 40,250 (18.3%) people are rural dwellers. Out of the total population 112,766 are females with a pregnancy rate of 3.7% which implies the total number of expected pregnant women in a given year is 8,152. According to the 2008/9 Gregorian calendar annual zonal report, the antenatal coverage in the study area was 71.5% [6-8].

### Participants

Three hundred sixty nine Antenatal care service users and service providers in eight public health facilities participated in the study.

### Study population

Sampled antenatal care service users at the public health facilities during the study period and purposively selected antenatal care providers (those providers who have more experience and providing ANC services in each facility).

### Study units

Individual antenatal care service users and antenatal care providers.

### Inclusion criteria

All pregnant women using ANC during data collection period and can give consent.

### Exclusion criteria

Pregnant women who were seriously sick and unable to respond to the questions, pregnant women who could not give consent (age is < 18 years, mentally impaired).

### Sample size and sampling technique

#### Sample size

The sample size was calculated using a single population proportion formula assuming, proportion of clients satisfaction on antenatal care they received (50%) which gives maximum sample size due to the absence of previous study. Considering 5% margin of error (d) and confidence level of 95% ( z α/2 = 1.96). Based on the above information a sample size was 384. However, since the target population was <10, 000 correction formula was applied which gives 360 and by adding 10% non response rate, the final sample size was 396.

#### Sampling technique

For the quantitative study all public health facilities providing antenatal care services were included. To achieve the desired sample size, the number of pregnant women selected from each centre was determined by a proportional to size allocation based on the average number of ANC users in the most recent quarterly report of each health facility (62 from Felege-Hiwot, 123 from Bahir Dar H/C, 44 from Tis-Abay, 48 from Abay-mado, 59 from Han, 20 from Meshenti, 21 from Zegie and 19 from Zenzelma Health centers). Individual study subjects at each health facility were selected by systematic random sampling during the data collection period until the required sample size at each health facility was obtained. The sampling interval (k = 3) was calculated by dividing the source population to the total sample size (396) and this interval was used in all facilities to select study subjects. The first client was selected by simple random sampling among the first three ANC service users in the sample frame.

For the qualitative component of the study, one antenatal care provider was selected in each health facility by purposive sampling (the provider who have more experience based on the number of years he/she work in the antenatal clinic and providing ANC services in the facility) and interviewed about the availability and adequacy of resources for antenatal care service provision.

#### Data collection instrument

Data were collected using a pre-tested structured questionnaire that was adopted from Population Council and USAID standards developed to measure the integration of family planning and other reproductive health services like antenatal care [9]. The questionnaire included socio-demographic characteristics, attributes of structure, process (both technical and interpersonal aspects) and outcome. A semi-structured open-ended interview guide and observation checklists for observation of antenatal care service provisions and structural attributes were also used.

### Study variables

The dependent variable for the study was quality of antenatal care measured by client satisfaction and with the independent variables include socio-demographic variables (age, educational status, ethnicity, religion, marital status, occupation, place of residence, monthly income), Sex of provider, frequency of ANC visits, Privacy during consultation, time of initiation of ANC, accessibility, waiting time, duration of consultation time and availability of resources.

### Data collection

Data on the types of services ANC attendees received were collected through interviews and chart reviews. Pregnant women were interviewed by trained female nurse graduates, on their exit from ANC clinics. Following interviews, further informed consent was requested to access medical records. Medical records of interviewed women were reviewed, on the same day, to collect further information on the care they received during their visit.

Data on structural- attributes of quality were collected by conducting resource inventory in each of the study health facilities. An inventory checklist was used to see if there was uninterrupted supply of required resources for the provision of comprehensive ANC services.

For the qualitative component, the two supervisors (B. Sc Nurses ) from health facilities not included in the study observed the way of history taking, physical examination diagnosis approach and its management to one in every four of the study subjects before the exit interview and after oral consent was obtained from both the provider and the client. In-depth interview with one antenatal care provider was also carried out in each health institution by the principal investigator.

### Data analysis

Descriptive statistics was computed to determine the extent to which recommended components of ANC services are provided to pregnant women attending ANC clinics in the study health facilities.

To assure the reliability of the instrument Cronbach’s alpha coefficient was calculated and it was 0.845. To assess the association of different independent variables with the outcome variable, bivariate analysis was carried out. And multiple logistic regression analysis was carried out to identify the most important predictors of client satisfaction and to control confounding effect. Only variables with p value of less than 0.05 in the bivariate analysis were included for multivariate logistic regression analysis. Forward variable entry method was employed. Qualitative data was categorized and analyzed thematically.

### Ethical considerations

The proposal was approved by Ethical Review Committee of college of public health and Medical sciences of Jimma University before the start of the study. Written consent was obtained from Bahir-Dar Special zonal health department and from the respective health facilities. All the study participants (both clients and providers) were informed about the purpose of the study and finally verbal consent was obtained before interview or observation. The respondents had the right to refuse participation or terminate their involvement at any point during the interview. The information provided by each respondent was kept confidential. Furthermore, report writing did not refer a specific respondent with identifiers.

## Results

### Socio-demographic characteristics of study participants

Among a total of 396 study subjects 369 responded to the interview with 93.2% response rate. The median age of the respondents was 25.0 ± 4.69 years (SD) with age range between 18–40 years. Three hundred thirty two (90.0%) of the respondents were married; whereas 31(8.4%) were single. Almost half of the respondents 175 (47.4%) had no formal education while 42 (11.4%) and 51 (13.8%) had secondary and beyond secondary level. Majority of the respondents 328 (88.9%) were Amhara followed by 15 (4.1%) Tigre, and 14 (3.8%) Awi ethnic groups, respectively. Three hundred forty (92.1%) of the respondents were orthodox Christians followed by Muslims 25 (6.8%). A little more than two-third of the respondents 257 (69.6%) were urban dwellers and almost half of the study participants 184 (49.9%) were house wives (unemployed).

### Structural attributes of quality

On inventory, all health facilities had functional weight scale, microscope, fetoscope and stethoscope but sphygmomanometer was not available in one health facility. Uristix for detection of glucose and protein in urine, Venereal Disease Research Laboratory (VDRL) and hemoglobin measurements were available only in two of the eight public health facilities included in the study. Penicillin was available in all health facilities but iron sulfate/folic acid was present only in one facility. Private ANC examination room was provided only in two health facilities. Antenatal care guideline and water to wash hands in the examination room was available in none of the facilities.

### Process attributes-Interpersonal aspects

Sound interpersonal relations contribute to effective health counseling and to a positive rapport with clients [10].Effective listening and communication are also important. Almost all 365 (98.9%) and 359 (97.3%) of respondents reported that the providers seem interested and there was no interruption by the provider during consultation respectively. The qualitative component of the study (by observation) also demonstrated that respectful and friendly greeting was offered for a total of 78 (81.2%) clients. However 105 (28.5%) of women reported that the door was not closed during consultation and 51 (13.8%) of the study women revealed that there were people other than the provider during consultation. This was again supplemented by the qualitative component as only two health facilities had private ANC rooms while the remaining facilities had multi- purpose rooms on observation.

One hundred nineteen (32.2%) and 26 (7.0%) of the respondents claimed that the procedures and the diagnosis were not explained for them respectively.

### Process attributes-technical aspects

During the present study140 (37.9%) women started their ANC visit within their first trimester, 180 (48.8%) and 43 (11.7%) of the women initiated their visits within 4-6 months and after 6 months of gestation respectively. There was significant association between educational status of women and time of initiation of antenatal care (X^2^ = 7.23, p = 0.007) and the same is true between residence and time of initiation of antenatal care (X^2^ =4.91, p = 0.027).

Advice about nutrition, family planning and Insecticide Treated bed net utilization was given for 221(59.9%), 151 (40.9%) and 130 (35.2%) women, respectively. In this study 316 (85.6%) and 304 (82.3%) of the women were asked and tested for HIV respectively. For majority 356 (96.5%) and 327(88.7%) of women gestational age and uterine height were measured, respectively. This was also supported by the finding of the qualitative method of data collection (Table 1) that revealed Gestational age was calculated for 91 (94.2%) pregnant women on observation.

**Table 1:** Services and procedures performed for ANC clients at the public health facilities in Bahir-Dar Special Zone, May2010

**Service/procedure (N = 369)**	**Frequency (n)**	**Percent (%)**
Blood pressure measured	336	91.1
Gestational age estimated	356	96.5
Uterine height measured	327	88.6
Blood group and Rh test 133		36.0
VDRL test	73	31.7
Hemoglobin test	113	30.6
Urine test for glucose and albumin	128	31.7
Iron/folic acid given	133	36.0
Fetal heart rate measured (N = 229)	181	79.0

### Outcome attributes

Client satisfaction was rated by 12 items each having five point Likert scale from strongly disagree (1) to strongly agree (5) as shown in Table 2 which has internal reliability (Cronbach’s α of 0.845). This shows that the items were internally consistent. To see the total score of each respondent, the points obtained from the 12 items by each respondent were computed. A respondent had a minimum 5 and a maximum of 60 points on ANC satisfaction score. Clients were categorized as not satisfied (if they score below the mean) or satisfied (if they score ≥ to the mean satisfaction score) .The mean score for client satisfaction on the ANC services received was 21.92 and 174 (47.7%) of the study women were scored less than the mean satisfaction score (not satisfied).

**Table 2:** Level of clients satisfaction on ANC services provided in public health facilities of Bahir Dar Special Zone North West Ethiopia, May 2010

**Item**	**Level of satisfaction on each item, n (%)**					
	**Strongly disagree**	**Dis agree**	**Uncertain**	**Agree**	**Strongly agree**	**Mean ± SD**
Provider’s greeting was good and in a friendly way(polite)	-	4(1.1)	32(8.7)	174(47.2)	159(43.1)	4.32 ± 0.677
Waiting time was fair	4(1.1)	20(5.4)	30(8.1)	214(58.1)	101(27.4)	4.05 ± 0.818
Waiting area was adequate & with seats	-	10(2.7)	45(12.2)	169(45.8)	144(39.0)	4.21 ± 0.76
The provider was easy to understand	-	1(0.3)	23(6.2)	198(63.7)	147(39.8)	4.33 ± 0.60
The cost incurred for the service was fair	-	3(0.8)	10(2.7)	146(39.6)	209(56.6)	4.52 ± 0.60
privacy during consultation was maintained	12(3.3)	44(11.9)	60(16.3)	144(39.0)	148(40.1)	3.8 ± 1.09
Provider perform the procedure with cleanliness and sanitation	-	6(1.6)	49(13.3)	160(45.0)	148(40.1)	4.24 ± 7.38
The antenatal clinic has clean latrine & adequate water supply	4(1.1)	65(17.6)	120(32.5)	150(40.7)	30(8.1)	3.37 ± 0.90
You feel that today you received full information about ANC.	-	23(6.2)	45(12.2)	183(49.6)	117(31.7)	4.07 ± 0.83
You want to continue the rest ANC visits in this health facility.	2(0.5)	2(0.5)	19(5.1)	170(46.1)	176(47.7)	4.40 ± 0.66
you recommend your relatives &others to attend their antenatal visit in this facility	-	3(0.8)	26(7.0)	180(48.8)	16(43.4)	4.35 ± 0.65
Generally you are happy with all the services you have got today.	-	5(1.4)	18(4.9)	168(45.5)	178(48.2)	4.41 ± 0.65

(N = 369).

The major reasons given by respondents for non-satisfaction with the over-all perceived quality of care received in the clinic were; absence of clean latrine and inadequate water supply, receiving incomplete information about ANC, inadequate waiting area and seats, absence of privacy, long waiting time and difficulty to understand the provider.

### Predictors of client satisfaction with antenatal care

To assess the association of different independent variables (socio-demographic, structure and process attributes of quality) with the outcome variable (client satisfaction), bivariate and multiple logistic regression analysis were carried out.

Based on the multiple logistic regression analysis, sex of the provider, time of initiation of ANC, privacy during consultation, frequency of ANC visit, duration of consultation time and explaining the procedure before ANC examination were predictor variables for client satisfaction on antenatal care (Table 3).

**Table 3:** Predictors of client satisfaction greater than the mean score among ANC attending pregnant women at eight health facilities in Bahir Dar Special Zone, North West Ethiopia, 2010

**Predictor variables for client satisfaction**		**Scored above the mean satisfaction score**	**Scored below the mean satisfaction score**	**Crude OR and CI**	**Adjusted OR and CI**
Sex of provider	Female	146	146	0.55(0.33,0.93)	2.56 (1.38,4.73)
	Male	27	49		1.0
Time of initiation of ANC	Within the first trimester	125	103	2.33(1.51,3.60)	2.11(1.26, 3.54)
	After the first trimester	48	92		1.0
Was the door closed (privacy)	Yes	142	121	0.357(0.22,0.58)	2.12(1.12, 3.70)
	No	31	74		1.0
Procedures explained	Yes	148	101	0.18(0.11,0.30)	5.47(3.10, 9.65)
	No	25	94		1.0
Frequency of ANC visit	New	56	104	2.39(1.56,3.56)	1.0
	Revisit	117	91		2.92(1.75, 4.88)
Duration of consultation time	<20 minutes	104	175	5.81(3.34,10.00)	1.0
	20-40 minutes	69	20		4.18(2.19, 8.00)

## Discussion

Even though the new WHO antenatal model recommends that the blood pressure and weight of a pregnant mother should be measured in each ANC visit [11], this study demonstrated that the blood pressure and weight of 33 (8.9%) and 17 (4.6%) of the study women were not measured, respectively. This would make the identification of pregnant women who need special care to be unlikely with subsequent follow up and management. According to Ethiopian Demographic and Health survey (EDHS) 2005 report, 43.7% of weight and 59.2% of blood pressure measurements of study mothers respectively were not taken in Amhara Region [6]. The difference b/n the findings of EDHS 2005 and the present study might be attributed to training of staffs and availability of equipment like blood pressure measuring machines.

Another study done in Tanzania showed, even though carrying out specific examinations revealed heterogonous picture, some of the examinations were done very regularly (weighing, auscultation of the fetal heart beat, and palpation of the fundus) for 99% of clients [10]. Workload of the health personnel, lack of awareness about components of antenatal care services (lack of training) or negligence might explain part of the difference with this study.

WHO recommended routine iron supplementation to all pregnant women and hemoglobin measurement only at 32 weeks (the third visit) unless there are clinical signs of severe anemia [12]. However, in this study hemoglobin measurement and checking conjunctiva for anemia were done for 113 (30.6%) and 226 (61.2%) of the pregnant women. Nearly two-third of the women hadn’t received iron/folic acid. Even though hemoglobin test is recommended only at 32 weeks, this practice is still rare as it was requested with other routine tests during the first visit regardless of her gestational age. This finding was also complemented by the qualitative component of the study which revealed that Iron sulfate/folic acid was available only in one health facility and ANC providers were requesting it usually for women who had signs of anemia. In addition, women were obliged to buy it in private pharmacies/drug venders.

The client’s perspective is very important because satisfied clients often are more likely to comply with treatment and continue to use primary health services. Thus, the dimensions of quality that relate to client satisfaction affect the health and well-being of the community [1]. This study revealed that only less than half,175 (47.7%) of the pregnant women scored above the mean satisfaction score (satisfied) with the ANC service provided at the eight public health facilities. Absence of clean latrine and inadequate water supply, receiving incomplete information about ANC, inadequate waiting area and seats, absence of privacy, long waiting time and difficulty to understand the provider were among the reasons behind this finding in the present study.

This was also supported by a study done in tertiary health institutions in Osun State, Nigeria and Irbid, North Jordan that showed 22.5% and 36% of the respondents, respectively were not satisfied due to too much wasting of time, no privacy because of students, too boring health talk and rude attitude of staff [1,13]. Another study in Holeta, central Ethiopia also support this finding as it revealed that most of the women in both FGDs agreed that over all their perception on quality is good but there are some problems associated with inadequate skilled professionals, manner of some health providers, and shortage of equipments and sometimes there is lack of privacy. One of the discussant stated that, “If you go to health institution you will find low quality of services and lack of respect and mistreatment from some of the health care providers” [14].

The probable reason that high proportion of study women were not satisfied in this study might be related to lack of qualified health professionals like midwife nurses, lack of training for providers and absence of ANC guidelines in health facilities. This was also supplemented by the qualitative component of the study as no ANC guideline was found in the eight public health facilities and no service provider had training on ANC except that one of them had trained on emergency obstetrics care and one with prevention of mother to child transmission.

Client satisfaction with the ANC services received was influenced by the sex of the provider and privacy during consultation. Respondents who received ANC from female providers were about three times more likely to score above the mean satisfaction score than those who received from male providers [OR = 2.56, 95% CI = 1.38, 4.73]. Respondents whose privacy was maintained were about two times more likely to score above the mean satisfaction score than those whose privacy was not maintained [OR = 2.12, 95% CI = 1.12, 3.70]. This might be due to loss of freedom to discuss about their issues with male provider and presence of other person in the ANC room during consultation.

In addition women’s satisfaction with the care received was also influenced by the frequency of ANC visit. Those respondents who visited the ANC clinic for two or more times were about three times more likely to be satisfied than those who visited only once [OR = 2.92, 95% CI = 1.75, 4.88]. This could be due to the reason that expectations of women as well as their perception would largely depend on their knowledge about the expected care, which may be dependent on previous experience.

The results of this study might be biased as health providers might make an extra effort to give their better quality of service on the days that the research team visits the health facility and in spite of adequate information and reassurance, women may feel that the study was an audit process conducted by higher authority and thus responded in favor of the health facility. Apart from these excluding the private and the not-for- profit organizations in the study, interviewing only one ANC provider at each health facility and failure to assess the “presence of a birth preparedness and complication readiness plan’ for each of the study participants can also be taken as the limitation of this study.

## Conclusion

Specific examinations revealed heterogonous picture where some of the examinations were done very regularly while others were missed for most of the clients. In addition, no provider was trained about ANC specifically and there was no supportive (Table 1) supervision during the last six months preceding the study. Moreover, more than half of the study women were not satisfied with the quality of ANC they received. Independent predictors of level of satisfaction with the service were sex of the ANC provider, privacy during consultation, frequency of ANC visit, time of initiation of ANC, explaining the procedure during ANC, and duration of consultation time.

Efforts targeted at reducing maternal mortality can only be successful if health care providers make sure that the ANC they provide are attractive and satisfactory to their clients. This study highlighted that attention should be given to the provision of adequate information about ANC, keeping privacy during consultation, avoiding missed opportunities and availability of all the necessary standard guidelines and resources are helpful means for affecting ANC care favorably.

## Competing interests

The authors declare that they have no any financial or non-financial competing interests (political, personal, religious, ideological, academic, intellectual, commercial or any other).

## Authors’ contributions

TE was involved in the conception, design, analysis and interpretation, report and manuscript writing. MW and YK had been involved in the design, analysis and interpretation of the data, and report and manuscript writing. All authors read and approved the final manuscript.

## Pre-publication history

The pre-publication history for this paper can be accessed here:

http://www.biomedcentral.com/1472-6963/13/443/prepub
